# Stability of the Mitigating Effect of Students’ Perceived Teacher Enthusiasm on Class-related Boredom: Moderating Role of Boredom Proneness and Perceived Task Difficulty

**DOI:** 10.3390/ijerph17082645

**Published:** 2020-04-12

**Authors:** Chen Wang, Yunjun Hu, Xia Zhang, Jing Wang, Guangli Cui, Guanyu Cui

**Affiliations:** 1 Faculty of Psychology, Beijing Normal University, Beijing 100875, China; c201.wang@connect.qut.edu.au; 2 School of Psychology and Counseling, Faculty of Health, Queensland University of Technology, Brisbane 4095, Australia; 3 Oujiang College, Wenzhou University, Wenzhou 325035, China; hyunjun@126.com; 4 Department of Nursing, Henan Medical College, Zhengzhou 451191, China; zx9218@126.com; 5 Department of Education, Xinzhou Teachers University, Xinzhou 034000, China; hndxwjzrj@163.com; 6 Education Science College, Xuchang University, Xuchang 461000, China; xyzcui@163.com; 7 Department of Psychology, School of Education, Wenzhou University, Wenzhou 325035, China

**Keywords:** perceived teacher enthusiasm, class-related boredom, perceived task difficulty, stability, moderating role

## Abstract

The aim of the current study was to explore the stability of the mitigating effect of students’ perceived teacher enthusiasm on class-related boredom and the moderating role of boredom proneness and perceived task difficulty in such effect. A total of 984 students from five universities in China participated in the study. Questionnaires on class-related boredom, perceived teacher enthusiasm, boredom proneness, and perceived task difficulty were used to measure the respective variables. Results showed that boredom proneness and perceived task difficulty significantly moderated the relationship between perceived teacher enthusiasm and class-related boredom. Moreover, when considering perceived task difficulty, boredom proneness became silent in the moderating path between perceived teacher enthusiasm and class-related boredom. Even so, the mitigating effect of students’ perceived teacher enthusiasm on class-related boredom was stable in students with different levels of boredom proneness and perceived task difficulty. The implications for learning and teaching are discussed.

## 1. Introduction

Classrooms are not neutral spaces, but full of emotions; such class-related emotions are at the core of teaching and learning [1,2]. Class-related boredom is a negative emotion widely experienced by students, which may affect their learning process and outputs as well as health [3]. The latest research has shown that students’ perceived teacher enthusiasm negatively predicted their class-related boredom significantly, which suggests that increasing teacher enthusiasm may be an efficient way to mitigate students’ class-related boredom [4,5]. In the theories of boredom antecedents and related empirical research, individuals’ boredom proneness and perceived task difficulty were considered to be important factors influencing boredom in specific situations [6,7]. Cui et al. confirmed the predicting effect of students’ perceived teacher enthusiasm on class-related boredom after controlling for the effects of boredom proneness and perceived task difficulty; however, neither of the studies confirmed a stable mitigating effect of perceived teacher enthusiasm on students’ class-related boredom among students with different levels of boredom proneness and perceived task difficulty [4,5]. Exploring the relationships among these variables could provide an important research basis for future interventional studies. Therefore, it is necessary to further investigate the stability of the effect of students’ perceived teacher enthusiasm on their class-related boredom and the moderating role of boredom proneness and perceived task difficulty in this effect.

### 1.1. Class-Related Boredom

Boredom is one of the most commonly emotions which can be experienced in many settings [3]. Class-related boredom refers to a negative and low physiological arousal emotion experienced by students in the process of learning in the classroom [3,8]. Different from the general academic boredom, class-related boredom is the experienced boredom in the classroom setting. Although general boredom is perceived as a high arousal state by some researchers [9], the control-value theory of achievement emotions and more research in recent decades suggested class-related boredom to be a low arousal state [3]. Class-related boredom is a negative emotion which involves dissatisfaction and low arousal, and causes irrelevant thoughts (i.e., daydreaming), temporal extension, and motivation to leave the boring situation [10,11,12]. The behavioral expression of this negative emotion mainly consists of sleepiness, yawning, flabby body posture, cold hands, and vacant eyes [3].

Class-related boredom is widely experienced by undergraduate students in the classroom. Mann and Robinson found that 59% of undergraduates reported experiencing boredom during half of the time during their lectures, and 30% reported that most or all of the time in their lectures was boring [13]. Pekrun et al. found that in the process of class-related learning, 42.2% undergraduates experienced boredom, which is higher than the rate of undergraduates experiencing emotions such as anxiety (28.0%), anger (19.3%), and hopelessness (13.6%) [3]. Tze and colleagues found that Chinese university students experienced more boredom in their classrooms than did their Canadian counterparts [14].

The influence of class-related boredom on students’ learning manifests itself, mainly, in the following aspects. Firstly, it was found to have effects on students’ class-related emotions, achievement goals, task value, self-efficacy in learning, learning engagement, use of learning strategies, and behavior problems [15,16,17]. Secondly, research found that students’ class-related boredom affected their achievement and performance [14,18,19]. Lastly, students’ class-related boredom was confirmed to have an influence on learning burnout [20], career aspirations [21], and lifelong learning [22]. Therefore, the effects of students’ class-related boredom on their learning and vocational development should not be underestimated; thus, investigating the antecedents of class-related boredom and identifying an efficient method to mitigate it is urgently required.

In the classroom environment, beside students’ individual antecedents, characteristics of teachers and teaching are important environmental antecedents of students’ class-related emotions [23]. In the view of control-value theory of achievement emotions, Pekrun suggested that important classroom environmental variables included instruction, value induction, autonomy support, goal structures/expectations, and achievement [24]. Goetz and Hall classified the antecedents of boredom into three categories: Individual (e.g., boredom proneness), environmental (e.g., monotony, isolation, or repetitive task), and related to the fit of individuals and environment (e.g., too hard or too easy task) [11]. Although some researchers suggested the importance of latent cognitive antecedents such as attention and hyperactivity disorder and impulsivity [25], current research has mainly focused on antecedents related to individual and environmental aspects.

### 1.2. Perceived Teacher Enthusiasm

The notion of teacher enthusiasm was developed and has evolved for decades. Initially, teacher enthusiasm was defined as enthusiastic teaching behaviors. Later, Kunter et al. defined teacher enthusiasm as teachers’ stable affection for subjects and teaching [26]. Recently, Keller, Goetz, Becker, Morger, and Hensley integrated an earlier definition of teacher enthusiasm with that by Kunter et al., proposing the definition of dispositional teacher enthusiasm, which included positive affect and positive emotional expressivity, and verifying the construct [27].

Teacher enthusiasm reflects a positive emotion and has been found to improve a range of students’ learning outcomes, such as school achievement, recall performance, and learning motivation [28,29]. However, only a few empirical studies have explored the relationship between teacher enthusiasm and students’ class-related boredom. For instance, in the Latin class setting, teacher enthusiasm was found to be one of the teaching variables highly related to students’ boredom [30]. Goetz and colleagues suggested teacher enthusiasm as one of teachers’ core teaching characteristics, and found that supportive presentation style, which included teacher enthusiasm, could significantly predict students’ class-related boredom [31]. Moreover, a group of researchers recently reported the direct and indirect relationships between teacher enthusiasm and students’ class-related boredom [4,5]. In a similar study, an outcome variable model of teacher enthusiasm was constructed, and an indirect predictive role of teacher enthusiasm on class-related boredom was supported [27]. In addition, students’ interest was considered a major antecedent of their boredom in theoretical research [7], and empirical research also found that interest could significantly predict class-related boredom [3,32].

Furthermore, some researchers suggested teacher enthusiasm as a core index of students’ perceived classroom environments [33] and instruction quality [30,31]. In fact, teacher enthusiasm has been known as a core index of instruction quality and teaching effectiveness for a long time, due to earlier educational studies [34,35,36]. In recent years, with the increasing research on students’ academic emotions as well as teacher emotions [23,37], some researchers have integrated related concepts of teacher enthusiasm and examined their effect on teachers’ teaching and students’ learning [38].

Although teacher enthusiasm may play an important role in mitigating students’ class-related boredom, few studies have investigated this relationship [4,5]. In terms of a theoretical basis, theories explaining the mitigating role of teacher enthusiasm on class-related boredom have been limited. Previous theories on the antecedents of boredom focused on boredom in the work environment, and the repetition of tasks and monotony of the environment were recognized as major antecedents of boredom [9]. Few theories have explained the antecedents of boredom in the classroom environment, especially focusing on the roles played by teachers [6]. Pekrun suggested that teacher enthusiasm may affect students’ achievement emotions through the mechanism of emotional contagion and observational learning in the control-value theory of achievement emotions [24]; however, no definite relationship between teacher enthusiasm and students’ class-related boredom is explained by this theory.

To the best of our knowledge, few studies have explored the relationship between teacher enthusiasm and class-related boredom in the domain of teacher emotions and academic emotions of students. Based on the control-value theory of achievement emotions and related results of empirical studies, we inferred that teacher enthusiasm may predict students’ class-related boredom.

### 1.3. Boredom Proneness

Although some researchers have defined boredom proneness, there is no widely accepted definition to date. Zuckerman, Eysenck, and Eysenck provided a definition for a similar concept, that is, boredom susceptibility as “an aversion to repetition, routine, and dull people, and restlessness when things are unchanging” [39]. Famer and Sundberg defined boredom proneness as “one’s connectedness with one’s environment on many situational dimensions, as well as the ability to access adaptive resources and realize competencies”. Boredom proneness was significantly related to depression, anxiety, stress, life satisfaction, and autonomy orientation [40,41].

In the classroom setting, students’ boredom proneness may be one of the major predictors of their class-related boredom. Empirical and theoretical evidence exists to support such association. For instance, students’ boredom proneness significantly predicted their scores on cognitive failure [42]. Based on the control-value theory of achievement emotions, students’ cognitive failure may affect their evaluation of control and value, and then induce class-related boredom [24]. Therefore, it could be concluded that students’ boredom proneness is associated with class-related boredom. Furthermore, boredom proneness was found to be predictive of the level of flow and mood monitoring, which may lead to class-related boredom [43]. Students with high boredom proneness were more likely to have internet addiction, but had a low tendency to engage in online learning [44]. More direct evidence was provided by a study on boredom in lecture, that students with a high level of boredom proneness reported higher levels of class-related boredom, displayed corresponding behaviors, used more boredom coping strategies, and were more likely to miss a lecture [13]. To sum up, boredom proneness is highly likely to be a core predictor of class-related boredom.

### 1.4. Perceived Task Difficulty

Perceived task difficulty has been found to affect academic boredom [6,45,46]. Specifically, Daschmann and colleagues found that students being over- or under-challenged in class-related learning were major predictors of their boredom in learning, and both variables have been confirmed as important precursors of boredom [6]. Tanaka and Murayama found that students’ perception of difficulty was associated with their boredom [45]. Asseburg and Frey found that the level of ability-difficulty fit of ninth-graders was significantly related to their boredom in a test, with students with a higher level of ability-difficulty fit having lower levels of boredom [46]. An explanation as to why high task difficulty and overload are associated with students’ boredom may be that high-difficulty tasks lead to excessive cognitive load and lack of fluency, which may decline students’ perceived value of the tasks and increase their experience of boredom in learning [45,47]. In contrast, a study found a decline in boredom with increasing difficulty [48], which seems to be inconsistent with the literature. To be noted, this study was conducted in the condition of easy tasks and students had a low level of anxiety. In such condition, an increase in difficulty indicated an increase in perceived challenge, and hence students perceive more control and value.

The framework of the control-value theory of achievement emotions suggests that students’ perceived control and value are proximate causes of achievement emotions, and that environmental variables affect emotions through the mediating role of students’ perceived control and value [24]. In accord with this, Fisher’s theory identified qualitative underload and overload as major causes of boredom [9]. According to the aforementioned studies and theories, we inferred that in learning tasks with medium difficulty, students’ increased perceived task difficulty will lead to a decline in their perceived control and value; therefore, negative emotions including boredom will be generated.

To sum up, students’ perceived task difficulty may be a major antecedent of boredom in various specific settings (e.g., boredom in learning and boredom in a test) [6,45]. However, there has been scarce research exploring the effect of task difficulty on students’ class-related boredom, and whether task difficulty can moderate the effects of antecedent variables on students’ class-related boredom.

### 1.5. Research Questions and Hypothesis

In previous theoretical and empirical studies on antecedents of boredom, boredom proneness and perceived task difficulty were conceived as important antecedent variables of specific and situational boredom. For example, in the control-value theory of achievement emotions and the study by Mann and Robinson, students’ boredom proneness and perceived task difficulty were important antecedent variables of class-related boredom [13,24]. Cui et al. found that after controlling for the effects of boredom proneness and perceived task difficulty, students’ perceived teacher enthusiasm predicted their class-related boredom negatively and significantly [4,5]. Based on the previous research by Cui and colleagues, the current study aimed to further explore whether boredom proneness and perceived task difficulty may moderate the relationship between perceived teacher enthusiasm and class-related boredom, and whether students’ perceived teacher enthusiasm can predict their class-related boredom significantly and stably among students with different levels of boredom proneness and perceived task difficulty. The present study aimed to explore the following two questions:

Question 1: Can students’ boredom proneness moderate the relationship between their perceived teacher enthusiasm and class-related boredom, and can perceived teacher enthusiasm predict class-related boredom stably and significantly among students with different levels of boredom proneness?

Question 2: Can students’ perceived task difficulty moderate the relationship between their perceived teacher enthusiasm and class-related boredom, and can perceived teacher enthusiasm predict class-related boredom stably and significantly among students with different levels of perceived task difficulty?

**Figure 1:**
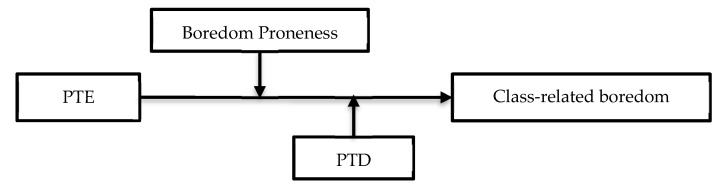
Predictive effect of perceived teacher enthusiasm (PTE) on class-related boredom: Moderating role of boredom proneness and perceived task difficulty (PTD).

Research population in the present study was college students. Although a number of research studies on teacher enthusiasm and boredom involved primary, middle, or high school settings, the present study with college students could investigate these variables and their associations in the setting of higher education. Results might provide significant practical implications to the teaching and learning of college students. Corresponding to the research questions, two research hypotheses were proposed. Hypothesis 1: Boredom proneness can moderate the relationship between perceived teacher enthusiasm and class-related boredom significantly, and students’ perceived teacher enthusiasm can predict their class-related boredom stably, negatively, and significantly among students with different levels of boredom proneness. Hypothesis 2: Students’ perceived task difficulty can moderate the relationship between perceived teacher enthusiasm and class-related boredom significantly, and students’ perceived teacher enthusiasm can predict their class-related boredom stably, negatively, and significantly among students with different levels of perceived task difficulty. Figure 1 shows the relationship indicated in the hypotheses.

## 2. Materials and Methods

### 2.1. Participants and Procedures

To test the research hypotheses, 984 students (73.0% female) at five colleges in Henan and Shanxi provinces aging from 17 to 24 years (mean = 20.08, standard deviation [SD] = 1.24) participated in this research. These participants were recruited from 21 class subjects across 19 majors in 15 departments, and they were in the first, second, or third school year (n = 567, 310, and 107, respectively). The present study was part of a large longitudinal survey; the questionnaire on boredom proneness was administered at two weeks after the beginning of a new term, and the remaining questionnaires at four weeks after the previous questionnaire. Each student in the study completed the survey only once, in relation to a single class. Teachers and students provided informed consent before the survey, which was conducted at the end of the class, and the questionnaires were collected immediately.

### 2.2. Measures

#### 2.2.1. Class-Related Boredom

Eleven-item class-related boredom subscale in the Achievement Emotions Questionnaire (AEQ) [48] was used to assess students’ class-related boredom in this study. Two example questions were “The lecture bores me” and “I think about what else I might be doing rather than sitting in this boring class”. Responses were indicated on a five-point Likert scale ranging from one (strongly disagree) to five (strongly agree). A higher aggregated score indicated a higher level of class-related boredom. Cronbach’s alpha was 0.94 in the current study.

#### 2.2.2. Perceived Teacher Enthusiasm

Three items (i.e., “Our teacher in this class teaches with enthusiasm,” “Our teacher in this subject enjoys teaching compared to other courses”, and “Our teacher in this class tries to inspire students about the subject”) from the study by Keller et al. [27], were used to assess perceived teacher enthusiasm. Responses were indicated on a five-point Likert scale ranging from one (not at all true of me) to five (very true of me). A higher aggregate score indicated a higher level of perceived teacher enthusiasm. Cronbach’s alpha was 0.87 for this construct in this study.

#### 2.2.3. Boredom Proneness

The 12-item Boredom Proneness Scale Short Form (BPS-SF) was adapted to measure boredom proneness [49]. Consistent with previous research [4,5], two items were deleted to adapt to the Chinese culture (i.e., “I find it easy to entertain myself” and “It seems that the same old things are on television or the movies all the time; it’s getting old”) Ten items were maintained. An example question: “In any situation I can usually find something to do or see to keep me interested”. Responses were indicated on a seven-point Likert scale ranging from one (strongly disagree) to seven (strongly agree). A higher aggregate score indicated a higher level of boredom proneness. Cronbach’s alpha was 0.69 for this scale in the present study.

#### 2.2.4. Perceived Task Difficulty

Two questions were included in the study to assess students’ perceived task difficulty, which were commonly used in previous research [45,50]. The two questions are “Today’s class was hard for me” and “Compared to other courses, today’s class was hard for me”. Responses to both questions are indicated on a five-point Likert scale ranging from one (not at all true of me) to five (very true of me). A higher aggregate score indicates a higher level of perceived task difficulty. Cronbach’s alpha was 0.84 for this measure in the present study.

### 2.3. Statistical Analyses

All analyses were conducted using PASW statistics for Windows (Version 18, IBM Corp., Armonk, NY, USA). Firstly, we examined descriptive statistics (mean and SD) and intercorrelations of the variables. Subsequently, we tested the moderating effects of boredom proneness and perceived task difficulty on the relationship between perceived teacher enthusiasm and class-related boredom using a hierarchical regression analysis.

The data of perceived teacher enthusiasm, boredom proneness, and perceived task difficulty had been standardized. Tests on data in the present study showed that the assumptions of hierarchical regressions (e.g., normality, homoscedasticity, independence of errors of prediction, and linearity.) were met. Multicollinearity was tested by calculating tolerance for each independent variable and values of tolerance indicated that multicollinearity did not occur among the data.

## 3. Results

### 3.1. Means, SDs, and Intercorrelations of All Measures

Means, standard deviations, and variable intercorrelations are presented in Table 1. The significant intercorrelations provided a foundation for the further analysis of the moderating effects of boredom proneness and perceived task difficulty.

### 3.2. Moderating Effects of Boredom Proneness

To test Hypothesis 1, a hierarchical regression analysis was used to test the moderating role of boredom proneness on the relationship between perceived teacher enthusiasm and class-related boredom. After controlling for the effect of demographic variables on class-related boredom, the predictive effects of perceived teacher enthusiasm, boredom proneness, and the interaction between perceived teacher enthusiasm and boredom proneness on class-related boredom were tested individually. The results are presented in Table 2.

**Figure 2:**
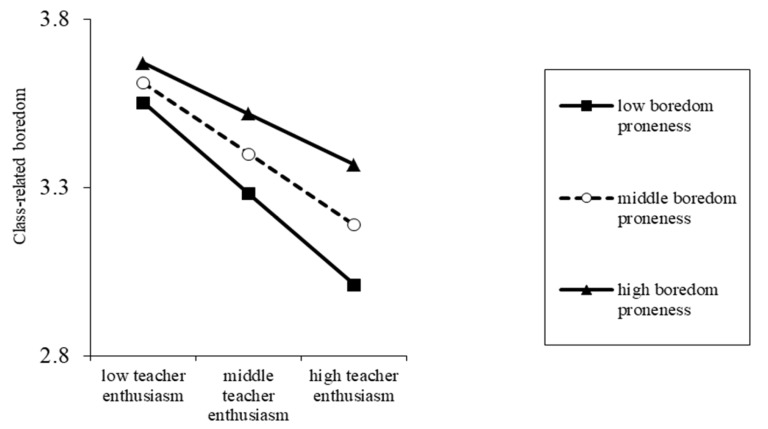
Predictive effect of perceived teacher enthusiasm on class-related boredom among college students with high and low boredom proneness.

As shown in Table 2, in the first step, major, subject, and age (among demographic variables) were significant predictors of class-related boredom (*p* < 0.01). In the second step, after controlling for the effects of demographic variables on class-related boredom, perceived teacher enthusiasm (*B* = −0.204, *p* < 0.001) and boredom proneness (*B* = 0.124, *p* < 0.001) were significant predictors of class-related boredom. In the third step, after controlling for the effects of demographic variables on class-related boredom, perceived teacher enthusiasm (*B* = −0.211, *p* < 0.001), boredom proneness (*B* = 0.118, *p* < 0.001), and the interaction between perceived teacher enthusiasm and boredom proneness (*B* = 0.058, *p* < 0.01) were significant predictors of class-related boredom. Thus, Hypothesis 1 was confirmed, as the results manifested that boredom proneness moderated the relationship between perceived teacher enthusiasm and class-related boredom. To be noted, perceived teacher enthusiasm had a significantly negative predicting effect on students’ class-related boredom. When perceived teacher enthusiasm increased, students’ class-related boredom could decrease. Furthermore, simple slope tests were used to test differences in the predictive effect of perceived teacher enthusiasm on class-related boredom between college students with high or low levels of boredom proneness (see Figure 2).

The results of simple slope tests are shown in Figure 2. High or low boredom proneness refers to the level of boredom proneness above or below one SD of the mean, respectively. Similarly, high or low teacher enthusiasm refers to the level of teacher enthusiasm above or below one SD of the mean, respectively. For the students with high levels of boredom proneness, the mitigating effect of perceived teacher enthusiasm on class-related boredom was relatively weak (simple slope = −0.153, *t* = −5.141, *p* < 0.001). In comparison, for the students with low levels of boredom proneness, the mitigating effect of perceived teacher enthusiasm on class-related boredom was relatively strong (simple slope = −0.269, *t* = −8.061, *p* < 0.001). In summary, although boredom proneness significantly moderated the relationship between perceived teacher enthusiasm and class-related boredom, the mitigating effects of perceived teacher enthusiasm on class-related boredom were stable and significant.

### 3.3. Moderating Effects of Perceived Task Difficulty

To test Hypothesis 2, a hierarchical regression analysis was used to test the moderating role of perceived task difficulty between perceived teacher enthusiasm and class-related boredom. After controlling for the effects of demographic variables and boredom proneness on class-related boredom, the predictive effect of perceived teacher enthusiasm, perceived task difficulty, and interaction between perceived teacher enthusiasm and perceived task difficulty on class-related boredom were tested individually. The results are presented in Table 3.

**Figure 3:**
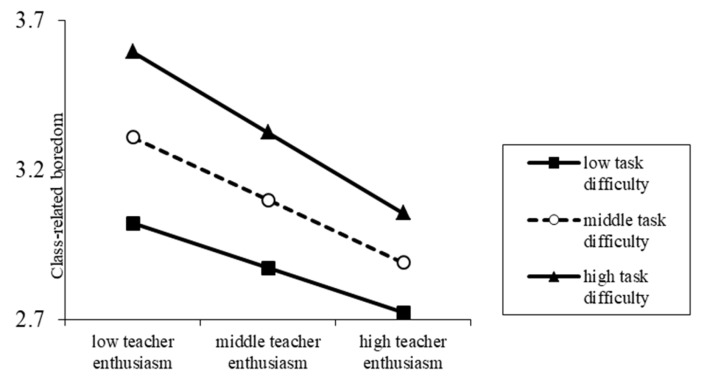
Prediction effect of perceived teacher enthusiasm on class-related boredom among college students with high and low perceived task difficulty.

The results of simple slope tests are shown in Figure 3. Consistent with the previous simple slope tests, perceived task difficulty and teacher enthusiasm were divided into high, medium, and low levels based on the scores above or below one SD of the mean. In the condition of high task difficulty, the mitigating effect of perceived teacher enthusiasm on class-related boredom was relatively strong (simple slope = −0.269, *t* = −8.574, *p* < 0.001). In comparison, the mitigating effect was relatively weak in the condition of low task difficulty (simple slope = −0.148, *t* = −5.068, *p* < 0.001). In summary, although perceived task difficulty significantly moderated the relationship between perceived teacher enthusiasm and class-related boredom, the mitigating effects of perceived teacher enthusiasm on class-related boredom were stable and significant.

### 3.4. Comparison of the Moderating Effects of Boredom Proneness and Perceived Task Difficulty

To further test the moderating effects, an additional hierarchical regression was conducted, which considered both boredom proneness and perceived task difficulty. The results are presented in Table 4. After controlling for the effect of demographic variables in Step one and the predictive effects of perceived teacher enthusiasm on class-related boredom in Step two, the moderating effects of boredom proneness and perceived task difficulty were tested in Step three and four, respectively. The significant moderating effect of boredom proneness showed in Step three turned to be non-significant after including perceived task difficulty in Step four (*p* = 0.102). Meanwhile, the moderating effect of perceived task difficulty is still significant even after controlling for the boredom proneness (*p* = 0.005). Comparison between the models tested in Step three and four showed that perceived task difficulty was a more direct and important moderator than boredom proneness between perceived teacher enthusiasm and class-related boredom.

## 4. Discussion

### 4.1. Moderating Effect of Boredom Proneness

According to the results of the present study, after controlling for the effects of demographic variables, boredom proneness played a significant moderating role in the relationship between students’ perceived teacher enthusiasm and class-related boredom. Perceived teacher enthusiasm was a relatively weaker predictor on class-related boredom among students with a high level of boredom proneness compared to those with a low level of boredom proneness.

The present results showing the moderating effect of boredom proneness on the relationship between perceived teacher enthusiasm and class-related boredom are consistent with other studies [13,40]. For instance, Farmer and Sundberg suggested that boredom proneness as a trait could moderate individuals’ boredom experience in specific settings [40]. They found that, compared to students with low boredom proneness, students with high boredom proneness had a higher level of boredom emotions and related behaviors. Mann and Robinson found that boredom proneness was an important antecedent of college students’ class-related boredom, and students with a high level of boredom proneness reported spent less time in lectures and missing more lecture time [13]. In addition, they found that compared to students with low boredom proneness, students with high boredom proneness used more coping strategies for boredom, and spent more time playing mobile phone games, sending text messages, and making shopping lists, and tended to “switch off,” write notes to others, daydream, and decide not to attend the next lecture [13]. Finally, Liu and colleagues found that students’ total scores and scores on all sub-scales of the multi-dimensional state boredom scale were positively and significantly correlated with their scores on boredom proneness [51]. According to these results, students’ boredom proneness may have important effects on their class-related boredom, especially for those with high boredom proneness levels, who easy experience boredom and find it hard to mitigate it. Therefore, boredom proneness may weaken the mitigating effect of perceived teacher enthusiasm on students’ class-related boredom.

### 4.2. Moderating Effect of Perceived Task Difficulty

Perceived task difficulty is an important task or environmental variable that may affect class behaviors and emotions of students [6]. In the theories on antecedents of boredom, earlier research suggested perceived repetitiveness, monotony, and low difficulty of tasks as major causes of boredom [3,9]. Subsequently, it was suggested that students’ perceived low or high task difficulty were causes of their class-related boredom, and those with perceived middle task difficulty may experience minimal boredom [6,45,52]. Furthermore, it was easier to elicit class-related boredom in settings with high task difficulty than in those with low difficulty [45].

Based on the previous contradictory findings, the present study explored the predictive role of students’ perceived task difficulty on their class-related boredom, and found that perceived task difficulty positively and significantly predicted class-related boredom, which is consistent with previous theories [24,52] and empirical results [6,45]. According to the control-value theory, the cause of achievement emotion is probably students’ perceived control and value, and environmental variables influent boredom through affecting students’ perceived control and value [3]. Higher perceived task difficulty results in a decline in students’ perceived control and value, and hence, boredom will be increased. Although previous theories and researchers considered that both low and high task difficulty were important antecedents of class-related boredom, empirical findings showed that high task difficulty had more serious effects on class-related boredom than did low task difficulty [6,7]. In factual educational settings, most classes in higher education are above the middle level of difficulty. The present study was conducted among college students and the actual learning tasks for them are usually medium or high.

The results of the present study showed that students’ perceived task difficulty moderated the relationship between perceived teacher enthusiasm and class-related boredom. Specifically, the perceived teacher enthusiasm of students who perceived high task difficulty had a stronger mitigating effect on their class-related boredom compared to students who perceived low task difficulty. This effect may due to the learning settings in college where most learning tasks involve middle or high difficulty. Compared to the students in primary or middle schools who perceived low task difficulty and experienced low level of boredom, college students who perceived high task difficulty experienced a higher level of boredom, thus providing enough space for mitigating class-related boredom. Therefore, perceived teacher enthusiasm had a stronger negative predictive effect on class-related boredom among students who perceived high task difficulty than among those who perceived low task difficulty. In summary, the current study showed that students’ perceived teacher enthusiasm had a negative and significant mitigating effect on their class-related boredom in most classes with middle task difficulty. It was difficult to find classes with a very high or very low task difficulty, so that perceived teacher enthusiasm would have no mitigating effects on class-related boredom in these extreme situations.

Interestingly, the comparison between the models with the two moderating variables suggested that when considering perceived task difficulty, boredom proneness became silent in the moderating path between perceived teacher enthusiasm and class-related boredom. Statistically, the moderating effect by boredom proneness could be mainly explained by perceived task difficulty. This means that the moderating effect of perceived task difficulty on the association is more direct than boredom proneness. This is a new finding and has not been explored in previous literature. However, the control-value theory could provide a possible explanation to this novel finding and indirect empirical evidence has existed in previous literature. According to the control-value theory, individual characters, such as boredom proneness, work on class-related boredom by affecting students’ perceived control and value [3], while perceived task difficulty can determine students’ perceived control and value [45]. Consistently, basic need satisfaction was found to matter more than personality in affecting students’ engagement and boredom [20]. To sum up, the effect of boredom proneness on boredom in a specific setting (e.g., class-related boredom) is less than setting-related variables (e.g., perceived task difficulty). Boredom proneness is a personality trait which is not easily changed across settings [43], whereas perceived task difficulty, perceived teacher enthusiasm, and class-related boredom are variables easily affected by learning settings [9]. Therefore, perceived task difficulty contributes more, compared with boredom proneness, to moderating the association between teacher enthusiasm and class-related boredom.

### 4.3. Negative and Stable Predictive Effect of Perceived Teacher Enthusiasm on Class-Related Boredom

The present study showed that although college students’ boredom proneness and perceived task difficulty moderated the relationship between perceived teacher enthusiasm and class-related boredom, perceived teacher enthusiasm still had a stable, negative, and significant predictive effect on class-related boredom among students with various levels of boredom proneness and perceived task difficulty. That is to say, although both boredom proneness as a trait antecedent variable and perceived task difficulty as an environmental antecedent variable had important effects on college students’ class-related boredom, their perceived teacher enthusiasm had a widespread, stable, and significant mitigating effect on class-related boredom. When increasing perceived teacher enthusiasm, students’ class-related boredom could decrease accordingly across various learning subjects.

### 4.4. Practical Implications

Considering learning and teaching practice, the present study confirmed the stable and widespread effect of teacher enthusiasm on students’ class-related boredom. Based on these results, educational researchers, administrators, and college teachers should pay more attention to the dampening effects of teacher enthusiasm on students’ class-related boredom. Therefore, more programs and measures for the cultivation of teacher enthusiasm should be implemented for teachers and pre-teachers, such as improving wages and treatment, and providing a better working environment and more humanistic care for teachers. In addition, it is important to train teachers to naturally and truly express their enthusiasm for their subjects and teaching before their students.

The present study showed that the dampening effect of teacher enthusiasm on class-related boredom was weaker among students with high boredom proneness, compared to those with low boredom proneness. Therefore, before intervening to decrease students’ boredom, for best results, it is necessary to measure their level of boredom proneness, and offer different intervention programs based on grouping according to their level of boredom proneness. For the students with low level of boredom proneness, increasing teacher enthusiasm could make a great difference. However, for the students with high level of boredom proneness, additional teaching strategies need to be considered to improve their learning effectiveness.

Compared to boredom proneness, which is difficult to change, there is a larger space for controlling for course difficulty. Results of the present study showed that the dampening effect of teacher enthusiasm on class-related boredom was stronger among students who had a high level of course difficulty, compared to those with a low level of course difficulty. Moreover, the moderating effect of perceived task difficulty between perceived teacher enthusiasm and class-related boredom is more direct than boredom proneness. Usually, college students face a medium or high level of learning challenge leading to relatively high levels of perceived course difficulty, which is one of the important antecedents of their class-related boredom. For the challenging courses, promoting teacher enthusiasm is an efficient and easy-to-implement intervention route to decrease students’ class-related boredom.

### 4.5. Limitations and Future Research Directions

The present study had some limitations. Firstly, the study was based on cross-sectional data and hence, it was difficult to draw causal inferences. Future research should further examine the relationships among these variables using longitudinal data. Secondly, the study focused on the college students. Findings might not apply for younger students in primary, middle, or high schools who typically face simpler learning tasks. Thirdly, the data were based on students’ perceptions and self-report measures, and other sources of data such as observation or video analysis were not included. Therefore, to some extent, the stringency of the results of the present study is relatively low. Future research should, as far as possible, use multi-source data to generate stronger evidence. However, some researchers consider students’ perceptions and self-report measures of class, teachers, teaching, and learning to be reliable.

## 5. Conclusions

To sum up, the current study suggested that boredom proneness and perceived task difficulty moderated the relationship between perceived teacher enthusiasm and class-related boredom; however, in general, perceived teacher enthusiasm could predict their class-related boredom stably and significantly among students with different levels of boredom proneness and perceived task difficulty.

## Figures and Tables

**Table 1:** Means, SDs, and intercorrelations of all measures.

	M	SD	1	2	3	4
1. Boredom Proneness	3.67	0.76	—			
2. Perceived Task Difficulty	2.76	1.00	0.12 **	—		
3. Perceived Teacher Enthusiasm	3.83	0.91	−0.16 **	−0.02	—	
4. Class-related Boredom	2.04	0.77	0.20 **	0.27 **	−0.30 **	—

Note. SD = standard deviation. ** *p* < 0.01.

**Table 2:** Moderating effect of boredom proneness between perceived teacher enthusiasm and class-related boredom (N = 984).

Variables	First Step		Second Step		Third Step	
	B	SEB	B	SEB	B	SEB
constant	3.893 ***	0.588	3.324 ***	0.558	3.433 ***	0.557
Step 1						
college	−0.070	0.068	−0.033	0.064	−0.047	0.064
department	−0.027	0.020	−0.009	0.019	−0.013	0.019
major	0.027 ***	0.006	0.023 ***	0.006	0.023 ***	0.006
grade	−0.015	0.049	−0.010	0.046	−0.015	0.046
subject	−0.007 **	0.002	−0.006 *	0.002	−0.006 **	0.002
gender old	−0.096 −0.064 **	0.060 0.024	−0.081 −0.050*	0.057 0.023	−0.076 −0.051 *	0.056 0.023
*R^2^*	0.038 ***					
Step 2						
PTE			−0.204 ***	0.023	−0.211 ***	0.023
Boredom proneness			0.124 ***	0.024	0.118 ***	0.024
R^2^			0.144 ***			
R^2^ change			0.106 ***			
Step 3						
PTE × Boredom proneness					0.058 **	0.021
R^2^					0.151 ***	
R^2^ change					0.007 **	

Note. PTE = perceived teacher enthusiasm; B = unstandardized regression coefficients; SEB = standard error of regression coefficients. * *p* < 0.05, ** *p* < 0.01, and *** *p* < 0.001.

**Table 3:** Moderating effect of perceived task difficulty between perceived teacher enthusiasm and class-related boredom (N = 984).

	First Step		Second Step		Third Step	
Variables	B	SEB	B	SEB	B	SEB
constant	3.573 ***	0.578	3.056 ***	0.547	3.108 ***	0.534
Step 1						
college	−0.052	0.066	−0.056	0.061	−0.065	0.061
department	−0.020	0.019	−0.012	0.018	−0.015	0.018
major	0.025 ***	0.006	0.025 ***	0.005	0.025 ***	0.006
grade	0.003	0.048	0.021	0.044	0.024	0.044
subject	−0.007 **	0.002	−0.007 **	0.002	−0.007 **	0.002
gender	−0.097	0.059	−0.011	0.055	−0.005	0.055
old	−0.055*	0.024	−0.039	0.022	−0.040	0.022
Boredom proneness	0.156 ***	0.024	0.106 ***	0.023	0.111 ***	0.023
R^2^	0.077 ***					
Step 2						
PTE			−0.204 ***	0.022	−0.209 ***	0.022
PTD			0.212 ***	0.023	0.226 ***	0.024
R^2^			0.212 ***			
R^2^ change			0.135 ***			
Step 3						
PTE × PTD					−0.061 **	0.020
R^2^					0.219 ***	
R^2^ change					0.007 **	

Note. PTD = perceived task difficulty. * *p* < 0.05, ** *p* < 0.01, and *** *p* < 0.001.

**Table 4:** Comparison of the moderating effects of boredom proneness and perceived task difficulty between perceived teacher enthusiasm and class-related boredom.

	First Step		Second Step		Third Step		Fourth Step	
Variables	B	SEB	B	SEB	B	SEB	B	SEB
constant	3.893 ***	0.588	3.548 ***	0.563	3.433 ***	0.557	3.175 ***	0.535
Step 1								
college	−0.070	0.068	−0.045	0.065	−0.047	0.064	−0.072	0.061
department	−0.027	0.020	−0.014	0.019	−0.013	0.019	−0.017	0.018
major	0.027 ***	0.006	0.023 ***	0.006	0.023 ***	0.006	0.025 ***	0.006
grade	−0.015	0.049	−0.025	0.047	−0.015	0.046	0.020	0.044
subject	−0.007 **	0.002	−0.006 *	0.002	−0.006 **	0.002	−0.008 **	0.002
gender	−0.096	0.060	−0.079	0.057	−0.076	0.056	−0.003	0.055
old	−0.064 **	0.024	−0.057	0.023	−0.051	0.023	−0.041	0.022
R^2^	0.038 ***							
Step 2								
PTE			−0.223 ***	0.023	−0.211 ***	0.023	−0.213 ***	0.023
R^2^			0.120 ***					
R^2^ change			0.082 ***					
Step 3								
BP					−0.118 **	0.024	0.108 ***	0.023
PTE × BP					0.058 **	0.021	0.034	0.021
R^2^					0.151 ***			
R^2^ change					0.031 **			
Step 4								
PTD							0.221 ***	0.024
PTE × PTD							−0.058 **	0.020
R^2^							0.221 ***	
R^2^ change							0.071 **	

Note. N = 984. BP = Boredom proneness. * *p* < 0.05, ** *p* < 0.01, and *** *p* < 0.001.
